# Wastewater treatment plant as a stable contributor affecting coastal ocean water viral communities

**DOI:** 10.1002/mlf2.70098

**Published:** 2026-08-17

**Authors:** Qiji Zhang, Zhanwen Cheng, Xiaotong Tang, Yuxi Yan, Yu Xia

**Affiliations:** ^1^ State Key Laboratory of Soil Pollution Control and Safety, School of Environmental Science and Engineering Southern University of Science and Technology Shenzhen China; ^2^ Guangdong Provincial Key Laboratory of Soil and Groundwater Pollution Control, School of Environmental Science and Engineering Southern University of Science and Technology Shenzhen China; ^3^ State Environmental Protection Key Laboratory of Integrated Surface Water‐Groundwater Pollution Control, School of Environmental Science and Engineering Southern University of Science and Technology Shenzhen China

## Abstract

Coastal viruses play a vital role in sustaining ecosystem functioning, and yet, the degree to which they are influenced by human activities remains poorly understood. Our study demonstrates that wastewater discharge fundamentally reshapes the coastal ocean virome, overriding its natural seasonal variability and highlighting a profound anthropogenic influence on the distribution, host associations, and ecological roles of viral communities. These findings advance our understanding of viral dynamics in coastal marine systems and establish a critical scientific basis for evaluating how human activities alter marine microbial ecology.

Viruses, the most abundant biological entities on Earth, play a crucial role in marine ecosystems^1^. They influence marine microbial communities through host metabolic reprogramming, cell lysis, and horizontal gene transfer^2^, and contribute to the biogeochemical carbon cycle by modulating host bacterial metabolism^3^. Conventional targeted methods such as quantitative PCR (qPCR) and flow cytometry are insufficient for characterizing complex viral communities. Given the extensive viral diversity, metagenomics is essential^4^, yet hampered by low viral nucleic acid content, necessitating concentration and purification to minimize contamination^5^, ^6^. Beyond these methodological issues, our understanding of the factors driving marine viral community composition remains limited. Although natural factors like geography, depth, temperature, and dissolved oxygen are known to shape viral communities^7^, ^8^, anthropogenic influences—particularly in human‐affected coastal areas—are poorly understood, motivating our investigation into these disturbances.

In this study, we sampled three offshore sites in Shenzhen: two sites (DMS and XC) located away from major urban centers and direct wastewater discharge points, and one site (SZW) situated at the outlet of a wastewater treatment plant (WWTP) within an urban area (Figure S1). Sampling was conducted in summer (June–August 2020) and winter (November 2020–January 2021). To examine how human activities influence coastal viral communities, we compared viral operational taxonomic units (vOTUs), their hosts, and auxiliary metabolic genes (AMGs) across regions and seasons, providing an overview of viral distribution and clarifying anthropogenic impacts.

In total, 38,017 viral contigs were identified in three sites among 6 months in summer and winter. Viral contigs were clustered into vOTU according to >95% identity and >80% coverage, and 27,112 vOTU were retrieved. Interestingly, although DMS and XC are geographically separated, they shared more vOTUs during the same season than each site shared across summer and winter seasons. In contrast, the SZW site shared more vOTUs across the two seasons, indicating that vOTUs were more stable in SZW rather than showing a seasonal fluctuation in DMS and XC. As the sampling only covered summer and winter of a single year, the potential influences of interannual variability and hydrological conditions (e.g., rainy and dry seasons) were not fully captured, which may constrain the generalization of the observed patterns.

Viral diversity indexes were calculated based on the abundance of vOTU in each site. The Shannon diversity, Simpson diversity, and the Pielou index of DMS and XC in summer were higher than those in winter, implying the effect of season on viral diversity. Specifically, at DMS, the Shannon index decreased from 3.03 in summer to 1.50 in winter, the Simpson index decreased from 0.92 to 0.53, and the Pielou index decreased from 0.45 to 0.23. Similarly, at XC, the Shannon index decreased from 3.10 to 2.50, the Simpson index decreased from 0.94 to 0.89, and the Pielou index decreased from 0.48 to 0.38 (Figure 1A). In contrast, SZW showed relatively minor seasonal variation (Shannon index: 3.05 vs. 2.98; Simpson index: 0.94 vs. 0.92; Pielou index: 0.52 vs. 0.48). Statistical comparisons (*t*‐test, *n* = 3) suggested significant seasonal differences at DMS and XC, but not at SZW. Bray–Curtis distance‐based non‐metric multidimensional scaling (NMDS) analysis showed that samples from SZW clustered closely, whereas samples from DMS and XC tended to cluster by season (Figure 1B). This might be explained by the fact that SZW is near the WWTP outlet, and this outfall is a stable factor for viral community. In contrast, in relatively less anthropogenically impacted coastal waters such as DMS and XC, viral communities are more likely to be influenced by natural environmental variability. Seasonal changes alter temperature and nutrient availability, thereby influencing viral replication, and seasonal succession of host microbial communities drives the change of viral communities^9^, ^10^.

**Figure 1:**
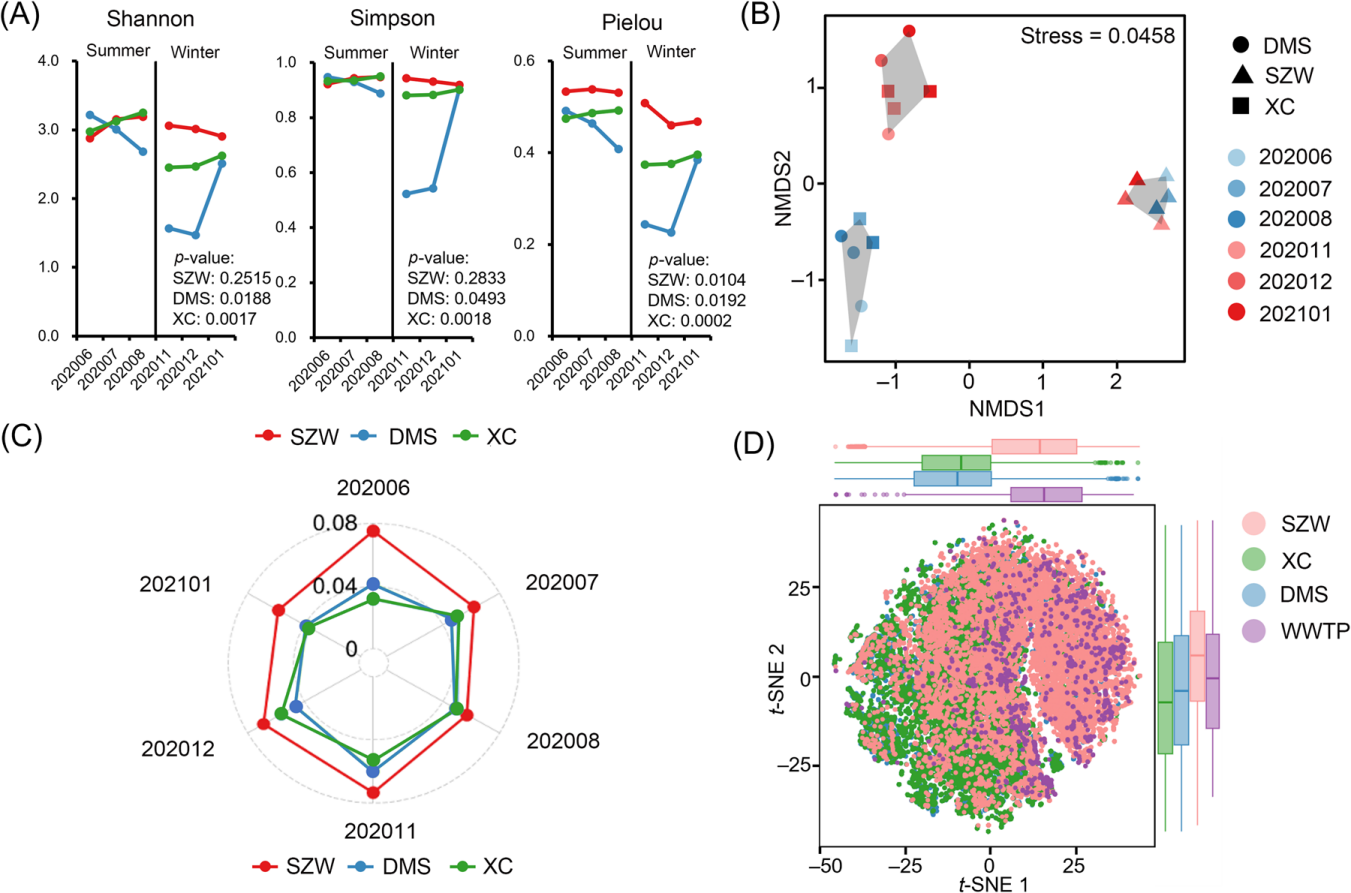
Comparison of the composition and diversity of coastal ocean viral communities across three sites (one wastewater‐impacted site [SZW] and two natural coastal sites [DMS and XC]). (A) Alpha diversity of viral communities across three sites and two seasons. The *p*‐values show the significant differences between summer and winter based on the *t*‐test. (B) Beta diversity of viral communities based on Bray–Curtis dissimilarity calculated from relative abundances of viral operational taxonomic units (vOTUs). (C) Proportion of lysogenic viruses in the viral communities across the three coastal sites. (D) *t*‐SNE analysis comparing viromes from three coastal sites and wastewater treatment plants (WWPTs) based on 5‐mer frequency.

Regarding viral lifestyles, the average proportion of lysogenic viruses was 0.66 at SZW, substantially higher than those at DMS and XC, both of which averaged 0.047. The proportion of lysogenic viruses at SZW was significantly higher than that at DMS and XC (Student's *t*‐test, *p* = 0.003 and 0.008, respectively; Figure 1C). This phenomenon may correlate with elevated environmental pressures in the WWTP effluent, where the oxidation‐reduction potential (ORP; Table S1) was higher at SZW (237 mV) than that at DMS (167 mV) and XC (199 mV). The WWTP effluent likely also contained residual chemical stressors, such as antibiotics and disinfectants^11^. Under such conditions, lysogenic viruses are preferentially harbored within bacterial hosts as a survival strategy. Additionally, by mediating horizontal gene transfer (HGT) through genome integration, lysogenic viruses enable bacterial hosts to acquire adaptive traits that enhance environmental stress resistance.

Among all sites, the majority of vOTUs were unclassified, with an average relative abundance of 65% across all samples. The dominant five families were *Kyanoviridae*, *Straboviridae*, *Mesyanzhinovviridae*, *Autographiviridae*, and *Zobellviridae* (Figures S2 and S3). Among the known viruses, DMS and XC showed a higher abundance of *Kyanoviridae*, accounting for approximately 20%. In contrast, *Casjensviridae*, *Mesyanzhinovviridae*, *Orlajensenviridae*, and *Peduoviridae* were more prevalent in SZW, with average relative abundances of 2.2%, 4.4%, 5.8%, and 2.5%, respectively. *Kyanoviridae* often infect cyanobacteria, and the natural scenic areas XC and DMS may have higher cyanobacterial biomass. The NO_3_^−^ concentration was much higher at SZW, with an average value of 10.31, compared with 1.42 at DMS and 1.50 at XC (Table S1). This elevated NO_3_^−^ level may promote the growth of heterotrophic bacteria and intensify microbial competition, thereby contributing to the suppression of phototrophic cyanobacteria. *Kyanoviridae* play a crucial role in the biogeochemical cycles of aquatic ecosystems by influencing the population dynamics and community structure of cyanobacteria^12^. The *Enterobacteriaceae* family is one of the primary hosts for *Casjensviridae*. The effluent from WWTP is rich in bacteria of intestinal origin, providing a suitable host for *Enterobacteriaceae*. Although seasonal differences were clearly reflected in community dissimilarity (Figure 1A), such patterns appeared to be less pronounced in the family‐level composition (Figure S3). This indicates that seasonal dynamics mainly occur at the vOTU level, whereas higher taxonomic groupings tend to remain relatively stable.

Except for viral taxonomic information, VPF‐tools also predicted the virus–host linkage. In all samples, approximately 97% of the vOTUs were predicted to infect bacteria, whereas only a small fraction were associated with eukaryotic and archaeal hosts, implying that most of the identified viruses in our studies were bacteriophages. This result aligns with the prevailing patterns observed in most ecosystems, where phages dominate^13^. However, it should be noted that our analysis focused on DNA viruses, and RNA viruses were not included; the viral community composition associated with RNA viruses may show a different pattern. In the family level, a total of 71 host families were predicted, and the top five bacteria with the highest viral abundance were *Enterobacteriaceae*, *Synechococcaceae*, *Bacillaceae*, *Flavobacteriaceae*, and *Rhodothermaceae* (Figure S4). The relative abundance of viruses associated with *Synechococcaceae* and *Bacillaceae* showed clear site‐specific differences. Viruses associated with *Synechococcaceae* were more abundant at DMS and XC, indicating stronger viral interactions with photosynthetic cyanobacteria at these two sites. In contrast, SZW showed a higher relative abundance of viruses associated with *Bacillaceae*, an important family within the *Firmicutes* phylum. WWTP effluents are commonly associated with chemical stressors, including residual disinfectants, antibiotics, and heavy metals, as well as physical pressures related to wastewater treatment processes, such as oxidative disinfection and mechanical shear^11^. These stress conditions may favor *Bacillaceae* hosts because their ability to form endospores confers resistance to heat, radiation, chemical stress, and drought^14^.

This study further used CRISPR‐based host prediction approaches to delineate virus–host interactions within the sampled ecosystems, successfully resolving linkages for 740 vOTUs (Figure S5). The predicted host affiliations revealed a striking dominance of *Proteobacteria*, accounting for 55.4% (*n* = 410) of all vOTU–host pairs. Within this phylum, *Gammaproteobacteria* (216 vOTUs) was the predominant class, followed by *Betaproteobacteria* (142 vOTUs), *Alphaproteobacteria* (38 vOTUs), and *Deltaproteobacteria* (14 vOTUs). *Gammaproteobacteria* and *Betaproteobacteria* are recognized as keystone taxa in nutrient cycling and organic matter degradation in the global ocean^15^. Beyond the *Proteobacteria*, host predictions identified substantial contributions from other phyla: *Actinobacteria* (13.8%, *n* = 102 vOTUs), *Firmicutes* (13.2%, *n* = 98 vOTUs), and *Bacteroidetes* (5%, *n* = 37 vOTUs).

The SZW site showed a higher abundance of viruses associated with these bacterial hosts, which was consistent with the stronger CRISPR signals detected in the associated microbial communities (Figure S5 and Table S2). Specifically, SZW contained 733 CRISPR sequences, substantially higher than DMS (98) and XC (85), indicating more frequent host–virus interactions in this anthropogenically impacted habitat. In summary, the viruses at the SZW site showed significant differences in terms of community composition, life cycle, and host relationships compared to those found at the DMS and XC sites.

This study identified a total of 12,125 potential viral AMGs from 884,437 viral protein sequences based on the protein annotation results from Virus Identification By iteRative ANnoTation (VIBRANT, Figure S6). The most involved metabolic pathway for these AMGs was carbon metabolism. The significant genes—*galE*, *UGDH*, *gmd*, *rfbE*, and *TSTA3*—each play a role in “O‐antigen nucleotide sugar biosynthesis,” a metabolic pathway classified under “glycan biosynthesis and metabolism”^16^. The synthesis of these components ensures that certain bacteria can specifically recognize O‐antigen receptors after being infected by bacteriophages, thereby protecting host cells from attacks by other phages.

Additionally, this study detected multiple genes involved in sulfur metabolism within nearshore marine viral sequences (Figure S6B), suggesting that nearshore marine viruses may also participate in the marine sulfur cycle. Notably, the gene *cysH* was found to be particularly abundant at the SZW sampling site; this gene encodes adenosine phosphosulfate reductase, a key enzyme in the assimilatory sulfate reduction pathway. This phenomenon is strongly associated with local environmental factors, as WWTP effluent is enriched with sulfate pollutants originating from multiple sources, including detergent residues (e.g., sodium dodecyl sulfate), industrial wastewater (such as textile dyeing and metal processing), and microbial breakdown of sulfur‐containing organic compounds. The influx of external sulfur has resulted in sulfur hotspots within SZW, compelling microorganisms to enhance sulfur assimilation metabolic activity to meet survival demands. Notably, viruses may facilitate host sulfur metabolism by carrying AMGs such as *cysH*, potentially improving conditions for their own replication.

To further confirm the impact of the WWTP on the SZW sampling site, this study collected effluent samples from the WWTP and compared the viruses found therein with those from SZW, DMS, and XC using Nanopore sequencing. Given the relatively higher error rate of Nanopore sequencing compared to short‐read platforms, *k*‐mer–based approaches were applied for viral identification and comparison, as they are less sensitive to sequencing errors and can help minimize potential platform‐related biases^17^, ^18^, ^19^. A total of 976 viral sequences longer than 10 kb were identified in the WWTP. The distribution of 5‐mers within these viral sequences suggests that the base distribution pattern of the virome from the WWTP closely resembles that of SZW, particularly in the *t*‐SNE 1 dimensions (Figure 1D). Moreover, by aligning the viromes from the WWTP with those from SZW, DMS, and XC, we found that 18.06% of the WWTP virome contributed to more than 80% of the virome in SZW (Figure S7). In contrast, the maximum contributions of the WWTP virome to DMS and XC were only 29.7% and 44.3%, respectively, and no WWTP‐derived virome contributed more than 80% to either site. This evidence suggests that the WWTP plays a significant role in shaping the viral community at SZW. However, as this study focuses on a single region and one WWTP, the generality of this pattern across different coastal systems remains uncertain. Multi‐regional studies are needed to further validate the universality of this finding.

These results underscore the substantial influence of anthropogenic activities in shaping coastal marine viromes. The proximity of SZW to the WWTP outlet suggests that effluent discharge introduces exogenous viral populations, which may dilute or even replace native viral communities, thereby altering ecosystem stability. Such anthropogenic viral inputs are of particular concern because WWTPs can harbor not only diverse bacteriophages but also viruses carrying pathogenic traits or antibiotic resistance genes, which could pose ecological and public health risks once released into coastal waters. Methodologically, the integration of Nanopore sequencing with *k*‐mer and *t*‐SNE analyses in this study highlights an effective approach for tracing viral sources and evaluating their contributions to marine communities.

This study provides comprehensive insights into the structure, functional potential, and host associations of viral communities in coastal marine ecosystems under varying anthropogenic influences. By comparing a wastewater‐impacted site (SZW) with two natural coastal sites, we demonstrate that viral diversity, composition, and metabolic potential are profoundly shaped by wastewater discharge. Unlike natural coastal viromes, which show pronounced seasonal dynamics, the virome at the SZW site remains relatively stable and closely resembles that of WWTPs, indicating that anthropogenic disturbance can override natural seasonal variability. Furthermore, *k*‐mer frequency analyses revealed strong similarities between the WWTP virome and that of the SZW site. These findings highlight the importance of accounting for viral responses to human activities when evaluating ecosystem health, and they provide a critical foundation for future investigations into the ecological of marine viruses in coastal environments.

## ETHICS STATEMENT

No human subjects, animal subjects, or endangered species were involved in this study. All sampling and experimental procedures complied with institutional and national guidelines for environmental research.

## Supporting information

Supporting File 1.

## Data Availability

The data supporting the findings of this study will be made available on request.
