# Supplementary material for: Wastewater treatment plant as a stable contributor affecting coastal ocean water viral communities
Source: mLife. 2026 Aug 17;5(4):525–9. doi: 10.1002/mlf2.70098 (PMC13481725; doi:10.1002/mlf2.70098)
Supplement: Supplementary file 1 [file MLF2-5-525-s001.docx]

**Supplementary Information**

**Wastewater Treatment Plant as A Stable Contributor Affecting Coastal Ocean Water Viral Communities**

**Qiji Zhang^a^, Zhanwen Cheng^a^, Xiaotong Tang^a^, Yuxi Yan^a^, Yu Xia^a,b,c,d^***

a Guangdong Provincial Key Laboratory of Soil and Groundwater Pollution Control, School of Environmental Science and Engineering, Southern University of Science and Technology, Shenzhen 518055, China

b State Environmental Protection Key Laboratory of Integrated Surface Water-Groundwater Pollution Control, School of Environmental Science and Engineering, Southern University of Science and Technology, Shenzhen 518055, China

c Guangdong Provincial Key Laboratory of Soil and Groundwater Pollution Control, School of Environmental Science and Engineering, Southern University of Science and Technology, Shenzhen 518055, China

d State Key Laboratory of Soil Pollution Control and Safety, School of Environmental Science and Engineering, Southern University of Science and Technology, Shenzhen 518055, China

*Corresponding author. E-mail address: xiay@sustech.edu.cn (Y. Xia)

Material and Methods

Site description and sampling procedure

A total of 20 L of ocean water was collected from three offshore sites (DMS: 22.596404°N, 114.305431°E; SZW: 22.523968°N, 113.99021°E; XC: 22.48195°N, 114.53784°E) along Shenzhen in June, July, and August 2020 (representing summer samples), and in November and December 2020 as well as January 2021 (representing winter samples). Among the three sampling sites, DMS and XC are natural coastal sites, whereas SZW is located adjacent to a WWTP outlet (Figure S1). Electrical conductivity, pH, and oxidation-reduction potential (ORP) were measured in situ using a 520M-01A multiprobe (Thermo). Five hundred milliliters of water were retained for the analysis of NH_4_^+^, NO^2-^, NO^3-^ using a UV-mini-1285 spectrophotometer (SHIMADZU). However, NH_4_^+^ and NO^2-^ were below the detection limit. The water samples were transported to the laboratory within 4 hours for downstream analyses. Abiotic data in this study are provided in Table S1.

Viral DNA extraction

Viral DNA was extracted and purified via chemical flocculation following established methods (John et al., 2011). Briefly, 20 L of ocean water was firstly pre-filtered with 1.6 μm Whatman FF/A filter to remove the large particles. The filtrates were filtered again to remove bacteria using a Millipore Express Plus filter (0.22 μm pore size, 142 mm diameter). FeCl_3_ was added to precipitate the viruses. After incubation at room temperature for 1 hour, the viral particles were collected using a 1.0 μm polycarbonate (PC) membrane filter placed atop a 0.8 μm Supor support filter. The viral precipitate was resuspended in ascorbic-EDTA buffer and concentrated using an Amicon® or Nanosep® centrifugal ultrafiltration device. Subsequently, 100 U·mL⁻¹ DNase I was added to remove bacterial and exogenous DNA, followed by the addition of 0.1 M EDTA and 0.1 M EGTA to halt DNase I activity. Viral DNA was extracted for downstream sequencing using the Wizard® PCR Preps DNA Purification Resin system (Promega, Madison, USA), with a ratio of 1 mL resin per 0.5 mL of concentrated sample. Universal bacterial 16S rRNA primers 515F/806R were employed in PCR assays to detect free and contaminating bacterial DNA. The extracted DNA was quantified with the Qubit™ dsDNA HS Assay Kit (Invitrogen, USA) and a NanoDrop™ ND-2000 spectrophotometer (Thermo Scientific, USA). Viral DNA was sequenced on an Illumina NovaSeq platform using a PE150 strategy with 350 bp insert libraries. 10 Gb data was targeted for each virome sample. Total sequencing output (base pairs and read counts per sample) is provided in Table S2.

Viral contig identification

The raw Illumina reads were first filtered for quality control (QC) using fastp (version 0.20.1) with default parameters (Chen et al., 2018). The post-QC reads were then de novo assembled using SPAdes (version 3.14.1) in “--meta” mode (Nurk et al., 2017). Only contigs longer than 5 kb were retained for downstream viral identification.

Three complementary approaches—VirSorter SOP (Roux et al., 2015), a vHMM-based virus detection pipeline (Paez-Espino et al., 2017), and a phylogenetic pipeline (Gudbergsdóttir et al., 2016)—were combined to identify viral contigs. To maximize viral signal recovery, all contigs identified by any of the three methods were merged. For the VirSorter SOP, contigs longer than 10 kb were analyzed with VirSorter, CheckV, and DRAMv. Of these, 37,843 contigs met the “keep” criteria and were retained as viral contigs. In the vHMM pipeline, genes were predicted with prodigal (Hyatt et al., 2010) (version 2.6.3) and annotated with hmmsearch (Eddy, 2011) (version 3.1b2) against Viral Protein Family Models (VPFs) (Paez-Espino et al., 2016) to identify viral genes, while against Pfam HMMs (33.1, --cut_nc mode) to identify other non-virus genes. All predicted genes were also compared to KEGG database (Kanehisa & Goto, 2000) (release 80.1) using DIAMOND (Buchfink et al., 2021) (version 0.9.22) with e-value of 1e-5 to identify non-virus genes. A contig will be considered as virus-like if it meets one of the three standards: 1) At least five genes in contigs are predicted to be VPFs, and these VPF genes account for more than 10% of total genes. Additionally, genes with non-virus KO terms should account for < 20% of all genes, while genes with non-virus Pfam hits should account for < 40% of all genes; 2) the genes on the contigs hit more VPFs than non-virus Pfam; 3) the VPF genes count for > 60% all predicted genes. For phylogenic based method, all assembled contigs were firstly aligned against NCBI nt database with e-value of 1e-5 using metablastn (Camacho et al., 2009); then a MEGAN-like Lowest Common Ancestor (LCA) algorithm was applied in taxator-tk (Droge et al., 2015) to get phylogenetic annotation of contigs. Meanwhile contigs were annotated with KRAKEN2 (Wood & Salzberg, 2014) with default parameters against default database. Contigs assigned to viruses by either approach was considered viral contigs. The vHMM and phylogenetic pipelines identified 1,760 viral contigs, and in total, 38,017 viral contigs, respectively.

Viral population and viral diversity

Viral contigs were clustered into vOTUs at >95% nucleotide identity and >80% coverage. The longest contig of each vOTU was selected as the representative for subsequent taxonomic analysis and host prediction. Coverage of each viral contig at each site was calculated as the number of virome reads mapped to the contig, normalized by contig length using Bowtie2 (Langmead & Salzberg, 2012). The relative abundance of each vOTU at each site was determined by the total coverage of the viral contigs within that vOTU. Shannon and Simpson diversity indices, Pielou’s evenness index, and beta diversity were calculated based on the top 150 most abundant vOTUs using the vegan package in R, as low-abundance vOTUs are prone to sequencing artifacts and may bias diversity estimates.

Viral taxonomic assignment

To predict taxonomic information of identified viruses, viral protein predicted by prodigal (Hyatt et al., 2010) were input into vConTACT2 (version 0.9.19) (Jang et al., 2019) to generate a gene-sharing network with prokaryotic viral RefSeq V201 using ClusterONE based on the protein sequence homology with default similarity cutoff. The taxonomic information of viruses could be determined by known RefSeq virus clustered inside VC and the taxonomy of vOTU could be represented by the longest viral contigs inside vOTU. However, this protein similarity-based method could only achieve taxonomy assignment of 178 vOTU, we further used a GCN based deep learning model (Shang et al., 2021) for viruses’ taxonomy classification.

Taxonomic profiling and virus-host prediction

Viral taxonomic profiling was performed using vpf-class (Pons et al., 2021), which classifies viruses based on viral protein families with default parameters. To further infer virus-host associations, we applied SpacePHARER (Zhang et al., 2021), a CRISPR spacer–based modular toolkit. Unlike viral protein family-based classification, CRISPR spacer matching provides direct evidence of past interactions between bacterial hosts and viruses. Therefore, SpacePHARER was used as a complementary approach to improve the confidence of virus-host prediction.

Specifically, all identified viral contigs were used to construct the targetSetDB, while a controlSetDBwas generated from the same contigs under default parameters to calibrate the reporting cutoff. The spacers_shmakov_et_al_2017 dataset provided by SpacePHARER was adopted as the querySetDB, as it contains more than 30,000 CRISPR spacer sets derived from bacterial genomes in the NCBI database (n = 580,383) with taxonomic identifiers (Shmakov et al., 2017). For each virus-host linkage, host taxonomy was determined using the lowest common ancestor (LCA) approach.

Identification of viral AMGs

Based on the annotation results generated by VIBRANT (Kieft et al., 2020), potential AMGs were predicted from viral sequences. VIBRANT integrates protein annotations from the KEGG, Pfam, and VOG databases to identify metabolism-related genes as candidate AMGs. To minimize false positives, CheckV (Nayfach et al., 2021) was further applied to exclude AMGs located on proviral regions.

Viral analysis in WWTP effluent

This study collected ultraviolet-treated effluent samples from the Futian WWTP in Shenzhen (114.1033°E, 22.5684°N) in January 2021, where three replicate samples were collected and combined into a single composite sample for sequencing analysis. The treated effluent is discharged directly into the ocean via an outfall pipeline, with its release point overlapping the SZW sampling sites. For these effluent samples, metagenomic material was obtained by filtration through 0.22 μm membranes. DNA extraction was performed using the FastDNA® SPIN Kit for Soil, followed by quantification with a NanoDrop spectrophotometer and Qubit 3.0 Fluorometer for Nanopore sequencing. Nanopore sequencing was conducted using an FLO-MIN 106 flow cell on a GridION X5 sequencer, with MinKNOW software managing both sequencing and basecalling steps.

For viral identification in Nanopore data, conventional protein annotation-based methods (e.g., vHMM and VirSorter) were unsuitable due to random insertion errors inherent to Nanopore sequencing. Instead, taxonomic annotation was performed using Centrifuge (Kim et al., 2016), which relies on k-mer frequency distributions rather than ORF prediction, thereby mitigating interference from sequencing errors. To assess the similarity between wastewater-derived viruses and coastal marine viruses, the 5-mer frequency profiles of all viral sequences from WWTP and viral contigs from the three sampling sites were calculated. And t-SNE dimensionality reduction was applied to the 5-mer frequency matrices to visualize sequence composition similarities, elucidating the potential influence of wastewater-derived viruses on coastal marine viral communities at the nucleotide level. All viral sequences identified in the wastewater effluent were mapped to quality-controlled virome datasets from the three coastal sampling sites using Bowtie2 (Langmead & Salzberg, 2012). The alignment results quantified the prevalence of wastewater-derived viral sequences across the marine samples.

| **Table S1 Abiotic data of samples used in this study** | | |  |  |  |  |  |
| --- | --- | --- | --- | --- | --- | --- | --- |
| **Sample** | **Time** | **Latitude** | **Longitude** | **pH** | **ORP (mv)** | **Conductive (μs/cm)** | **NO3- (μg/ml)** |
| dms202006 | 2020.6 | 22.596404N | 114.305431E | 8.36 | 141.9 | 1245 | 1.44 |
| dms202007 | 2020.7 | 22.596404N | 114.305431E | 8.17 | 115.7 | 1304 | 1.38 |
| dms202008 | 2020.8 | 22.596404N | 114.305431E | 8.16 | 151.7 | 1227 | 1.17 |
| dms202011 | 2020.11 | 22.596404N | 114.305431E | 8.32 | 194.1 | 1388 | 1.49 |
| dms202012 | 2020.12 | 22.596404N | 114.305431E | 8.5 | 182.8 | 1559 | 1.66 |
| dms202101 | 2021.01 | 22.596404N | 114.305431E | 8.56 | 213.1 | 1648 | 1.38 |
| szw202006 | 2020.6 | 22.523968N | 113.99021E | 6.67 | 265.7 | 1257 | 8.55 |
| szw202007 | 2020.7 | 22.523968N | 113.99021E | 6.63 | 186 | 1288 | 11.37 |
| szw202008 | 2020.8 | 22.523968N | 113.99021E | 6.62 | 198 | 1248 | 13.05 |
| szw202011 | 2020.11 | 22.523968N | 113.99021E | 6.5 | 247.8 | 1340 | 9.85 |
| szw202012 | 2020.12 | 22.523968N | 113.99021E | 7.29 | 254.4 | 1491 | 9.47 |
| szw202101 | 2021.01 | 22.523968N | 113.99021E | 7.65 | 268.1 | 1513 | 9.58 |
| xc202006 | 2020.6 | 22.48195N | 114.53784E | 8.35 | 292.2 | 1243 | 1.28 |
| xc202007 | 2020.7 | 22.48195N | 114.53784E | 8.3 | 84.1 | 1339 | 1.28 |
| xc202008 | 2020.8 | 22.48195N | 114.53784E | 8.2 | 41.9 | 1252 | 1.44 |
| xc202011 | 2020.11 | 22.48195N | 114.53784E | 8.37 | 180.1 | 1369 | 1.44 |
| xc202012 | 2020.12 | 22.48195N | 114.53784E | 8.41 | 233 | 1559 | 1.98 |
| xc202101 | 2021.01 | 22.48195N | 114.53784E | 8.55 | 363.9 | 1680 | 1.60 |

| **Table S2 Summary of sequencing data in this study** | | | | |  |  |  |  |  |  |  |
| --- | --- | --- | --- | --- | --- | --- | --- | --- | --- | --- | --- |
| **Sample** | **Time** | **Site** | **Total reads(m)** | **Total bases(g)** | **Assembled number  ( ≥1kb )** | **N50  (≥1kb )** | **Identified viral contig number** | **vOTU** | **Viral proportion** | **CRISPR sequence** | **Anti-CRISPR** |
| dms202006 | 2020.6 | dms | 79.49289 | 11.923933 | 106300 | 2734 | 575 | 569 | 11.52% | 6 | 0 |
| dms202007 | 2020.7 | dms | 99.111962 | 14.866794 | 125827 | 3036 | 1907 | 1879 | 14.59% | 13 | 0 |
| dms202008 | 2020.8 | dms | 109.949176 | 16.492376 | 133535 | 3072 | 2628 | 2589 | 18.14% | 29 | 0 |
| dms202011 | 2020.11 | dms | 82.886158 | 12.432924 | 115261 | 3293 | 2709 | 2652 | 23.00% | 20 | 0 |
| dms202012 | 2020.12 | dms | 85.616862 | 12.842529 | 112929 | 3300 | 2703 | 2605 | 26.32% | 21 | 0 |
| dms202101 | 2021.01 | dms | 79.271332 | 11.8907 | 91391 | 2960 | 1569 | 1523 | 22.57% | 9 | 0 |
| szw202006 | 2020.6 | szw | 120.188504 | 18.028276 | 170421 | 3282 | 731 | 716 | 4.64% | 64 | 2 |
| szw202007 | 2020.7 | szw | 115.50931 | 17.326396 | 122189 | 3634 | 3443 | 3342 | 26.36% | 146 | 6 |
| szw202008 | 2020.8 | szw | 109.723396 | 16.458509 | 120778 | 3832 | 3795 | 3701 | 26.56% | 126 | 1 |
| szw202011 | 2020.11 | szw | 108.881226 | 16.332184 | 114770 | 3628 | 3008 | 2889 | 24.74% | 129 | 6 |
| szw202012 | 2020.12 | szw | 109.489664 | 16.42345 | 129782 | 2904 | 2808 | 2672 | 24.46% | 111 | 1 |
| szw202101 | 2021.01 | szw | 108.424172 | 16.263626 | 104755 | 4053 | 3565 | 3479 | 26.38% | 157 | 3 |
| xc202006 | 2020.6 | xc | 44.996642 | 6.749496 | 57092 | 2160 | 181 | 177 | 14.55% | 3 | 0 |
| xc202007 | 2020.7 | xc | 50.388242 | 7.558236 | 80569 | 2569 | 965 | 946 | 12.95% | 11 | 0 |
| xc202008 | 2020.8 | xc | 77.476076 | 11.621411 | 112435 | 2420 | 763 | 737 | 11.15% | 12 | 0 |
| xc202011 | 2020.11 | xc | 66.274528 | 9.941179 | 105709 | 2719 | 1716 | 1637 | 23.79% | 16 | 0 |
| xc202012 | 2020.12 | xc | 76.31028 | 11.446542 | 100717 | 3237 | 2422 | 2331 | 27.18% | 23 | 0 |
| xc202101 | 2021.01 | xc | 89.416836 | 13.412525 | 130832 | 3180 | 2529 | 2439 | 22.17% | 20 | 0 |

Figure S1. Sampling sites of this study. SZW (22.52°N, 113.99°E) is situated near the Futian WWTP sewage outfall, whereas DMS (22.59°N, 114.30°E) and XC (22.48°N, 114.53°E) represent natural coastal waters. Photographs of the three sampling sites were taken by the authors.

Figure S2. Viral community composition across the three sampling sites.

Figure S3. The composition of coastal virus communities in the three sites at the family level.

Figure S4. Predicted virus hosts by VPF tools in the three sampling sites. (A) virus-host linkages at family level; (B) the relative abundance of the bacterial viruses.

Figure S5. Predicted virus hosts by CRISPR in the three sampling sites. (A) the number of vOTUs infected with bacteria and archaea; (B) the relative abundance of the viruses.

Figure S6. Function and abundance profiles of virus-encoded AMGs in the coastal marine waters. (A) the distribution of the major identified AMGs; (B) the abundance of the S cycle related genes.

Figure S7. Relationships of virome from WWTP and that from coastal marine water of this study: the matched proportion of virome from WWTP to that from three sampling sites.

**Supplementary References**

Buchfink, B., Reuter, K., & Drost, H. G. (2021). Sensitive protein alignments at tree-of-life scale using DIAMOND. *Nat Methods*, *18*(4), 366-368. https://doi.org/10.1038/s41592-021-01101-x

Camacho, C., Coulouris, G., Avagyan, V., Ma, N., Papadopoulos, J., Bealer, K., & Madden, T. L. (2009). BLAST+: architecture and applications. *BMC Bioinformatics*, *10*, 421. https://doi.org/10.1186/1471-2105-10-421

Chen, S. F., Zhou, Y. Q., Chen, Y. R., & Gu, J. (2018). fastp: an ultra-fast all-in-one FASTQ preprocessor. *Bioinformatics*, *34*(17), 884-890. https://doi.org/10.1093/bioinformatics/bty560

Droge, J., Gregor, I., & McHardy, A. C. (2015). Taxator-tk: precise taxonomic assignment of metagenomes by fast approximation of evolutionary neighborhoods. *Bioinformatics*, *31*(6), 817-824. https://doi.org/10.1093/bioinformatics/btu745

Eddy, S. R. (2011). Accelerated Profile HMM Searches. *Plos Computational Biology*, *7*(10). https://doi.org/10.1371/journal.pcbi.1002195

Gudbergsdóttir, S. R., Menzel, P., Krogh, A., Young, M., & Peng, X. (2016). Novel viral genomes identified from six metagenomes reveal wide distribution of archaeal viruses and high viral diversity in terrestrial hot springs. *Environmental Microbiology*, *18*(3), 863-874. https://doi.org/10.1111/1462-2920.13079

Hyatt, D., Chen, G. L., LoCascio, P. F., Land, M. L., Larimer, F. W., & Hauser, L. J. (2010). Prodigal: prokaryotic gene recognition and translation initiation site identification. *Bmc Bioinformatics*, *11*. https://doi.org/10.1186/1471-2105-11-119

Jang, H. B., Bolduc, B., Zablocki, O., Kuhn, J. H., Roux, S., Adriaenssens, E. M., Brister, J. R., Kropinski, A. M., Krupovic, M., Lavigne, R., Turner, D., & Sullivan, M. B. (2019). Taxonomic assignment of uncultivated prokaryotic virus genomes is enabled by gene-sharing networks. *Nature Biotechnology*, *37*(6), 632. https://doi.org/10.1038/s41587-019-0100-8

John, S. G., Mendez, C. B., Deng, L., Poulos, B., Kauffman, A. K. M., Kern, S., Brum, J., Polz, M. F., Boyle, E. A., & Sullivan, M. B. (2011). A simple and efficient method for concentration of ocean viruses by chemical flocculation (vol 3, pg 195, 2011). *Environmental Microbiology Reports*, *3*(6), 809-809. https://doi.org/10.1111/j.1758-2229.2011.00301.x

Kanehisa, M., & Goto, S. (2000). KEGG: kyoto encyclopedia of genes and genomes. *Nucleic Acids Res*, *28*(1), 27-30. https://doi.org/10.1093/nar/28.1.27

Kieft, K., Zhou, Z. C., & Anantharaman, K. (2020). VIBRANT: automated recovery, annotation and curation of microbial viruses, and evaluation of viral community function from genomic sequences. *Microbiome*, *8*(1). https://doi.org/10.1186/s40168-020-00867-0

Kim, D., Song, L., Breitwieser, F. P., & Salzberg, S. L. (2016). Centrifuge: rapid and sensitive classification of metagenomic sequences. *Genome Research*, *26*(12), 1721-1729. https://doi.org/10.1101/gr.210641.116

Langmead, B., & Salzberg, S. L. (2012). Fast gapped-read alignment with Bowtie 2. *Nature Methods*, *9*(4), 357-U354. https://doi.org/10.1038/Nmeth.1923

Nayfach, S., Camargo, A. P., Schulz, F., Eloe-Fadrosh, E., Roux, S., & Kyrpides, N. C. (2021). CheckV assesses the quality and completeness of metagenome-assembled viral genomes. *Nature Biotechnology*, *39*(5), 578-585. https://doi.org/10.1038/s41587-020-00774-7

Nurk, S., Meleshko, D., Korobeynikov, A., & Pevzner, P. A. (2017). metaSPAdes: a new versatile metagenomic assembler. *Genome Research*, *27*(5), 824-834. https://doi.org/10.1101/gr.213959.116

Paez-Espino, D., Eloe-Fadrosh, E. A., Pavlopoulos, G. A., Thomas, A. D., Huntemann, M., Mikhailova, N., Rubin, E., Ivanova, N. N., & Kyrpides, N. C. (2016). Uncovering Earth's virome. *Nature*, *536*(7617), 425. https://doi.org/10.1038/nature19094

Paez-Espino, D., Pavlopoulos, G. A., Ivanova, N. N., & Kyrpides, N. C. (2017). Nontargeted virus sequence discovery pipeline and virus clustering for metagenomic data. *Nature Protocols*, *12*(8), 1673-1682. https://doi.org/10.1038/nprot.2017.063

Pons, J. C., Paez-Espino, D., Riera, G., Ivanova, N., Kyrpides, N. C., & Llabrés, M. (2021). VPF-Class: taxonomic assignment and host prediction of uncultivated viruses based on viral protein families. *Bioinformatics*, *37*(13), 1805-1813. https://doi.org/10.1093/bioinformatics/btab026

Roux, S., Enault, F., Hurwitz, B. L., & Sullivan, M. B. (2015). VirSorter: mining viral signal from microbial genomic data. *Peerj*, *3*. https://doi.org/10.7717/peerj.985

Shang, J. Y., Jiang, J. Z., & Sun, Y. N. (2021). Bacteriophage classification for assembled contigs using graph convolutional network. *Bioinformatics*, *37*, I25-I33. https://doi.org/10.1093/bioinformatics/btab293

Shmakov, S. A., Sitnik, V., Makarova, K. S., Wolf, Y. I., Severinov, K. V., & Koonin, E. V. (2017). The CRISPR Spacer Space Is Dominated by Sequences from Species-Specific Mobilomes. *Mbio*, *8*(5). https://doi.org/10.1128/mBio.01397-17

Wood, D. E., & Salzberg, S. L. (2014). Kraken: ultrafast metagenomic sequence classification using exact alignments. *Genome Biol*, *15*(3), R46. https://doi.org/10.1186/gb-2014-15-3-r46

Zhang, R. S., Mirdita, M., Karin, E. L., Norroy, C., Galiez, C., & Söding, J. (2021). SpacePHARER: sensitive identification of phages from CRISPR spacers in prokaryotic hosts. Bioinformatics, 37(19), 3364-3366. https://doi.org/10.1093/bioinformatics/btab222
